# Admission Mechanisms for Scalable Federated Health Data Collaboration: Design and Proof-of-Concept Study

**DOI:** 10.2196/89685

**Published:** 2026-10-30

**Authors:** Enzo Fenoglio, Marzena Nieroda, Graca Carvalho, Malgorzata Smietanka, Dimitrios Kalogeropoulos, Philip Treleaven

**Affiliations:** 1 UCL Computer Science, University College London, London, England, United Kingdom; 2 UCL Global Business School for Health, University College London, Marshgate Building, 7 Sidings Street, London, England, E20 2AE, United Kingdom, 44 07739873221; 3 Departamento de Informatica, Universidade do Minho, Braga, Portugal; 4 Actuari, London, England, United Kingdom; 5 Global Health and Digital Innovation Foundation, London, England, United Kingdom

**Keywords:** federated learning, federated analytics, health data collaboration, federation admission, data governance, artificial intelligence, health informatics, federated computing

## Abstract

**Background:**

Many digital health and AI initiatives show promising results in pilot settings but do not progress to sustainable services. Pilots can operate within a predefined collaboration, whereas operational services must accommodate changes in participating organizations, available resources, and analytical capabilities while preserving established governance and technical arrangements.

**Objective:**

This study examines the pilot-to-service transition as a problem of governed federation evolution and evaluates whether Open Health can admit new forms of participation while preserving bounded capabilities, organizational sovereignty, and auditable decision evidence.

**Methods:**

Open Health applies federated computing (FC) as a governance model separated from the analytical runtime. A governed federation context is established before execution, participants receive holder-bound capabilities, and an admission gateway produces explicit ALLOW or DENY decisions before requested operations proceed. The proof of concept uses the Flower federated learning framework and the Pathology Modified National Institute of Standards and Technology (PathMNIST) dataset from the MedMNIST collection with an initial federation comprising hospitals A and B. Mode 1A introduces hospital C as a sponsored contributor permitted to submit locally computed training updates but not invoke the resulting federated model for inference. Mode 1B examines bounded AI-agent participation, where restricted results may be transformed through policy-authorized operations into derivatives whose consumption is governed independently from the source. Evaluation considers admission conformance, signed decision evidence, analytical effects of the additional contribution, and admission overhead.

**Results:**

The demonstrator admitted valid member operations and hospital C’s sponsored contribution (mode 1A) while denying model consumption outside hospital C’s capability, scope violations, holder-binding mismatch, and federation-envelope mismatch. Mode 1B extended the same bounded-governance pattern to AI-agent participation, admitting bounded inference and governed transformation but denying unauthorized source consumption and privileged governance access. Under matched training conditions, cancer-related macro recall increased from 50.2% in the A+B control to 67.4% in A+B+C (+17.2 percentage points), while noncancer macro recall changed from 85.5% to 86.3% (+0.8 percentage points). Recall increased from 19.5% to 39.9% for cancer-associated stroma and from 80.9% to 94.9% for colorectal adenocarcinoma epithelium. Median (IQR) full admission latency was 1.210 (1.141-1.333) ms for ALLOW, 1.195 (1.136-1.273) ms for capability-scope DENY, and 0.884 (0.837-0.921) ms for holder-binding DENY. Each evaluated request generated signed decision evidence recording the decision, reason, participant and request bindings, governance context, and related model run.

**Conclusions:**

Open Health demonstrates how a federated health-data collaboration can evolve through differentiated participation while preserving membership boundaries and bounded capability relations. The principal contribution is an executable governance boundary preserving capability relations and producing independently verifiable decision evidence separately from the federated-learning runtime. The analytical experiment showed that governed admission of an additional contributor produced a measurable effect on the intended cancer-related classes while preserving noncancer performance. The proof of concept establishes governance behavior under controlled conditions; clinical effectiveness and production-scale performance require further evaluation.

## Introduction

### Background

Digital health and clinical AI increasingly depend on access to diverse, multimodal, and representative data distributed across organizations and care settings. Models developed or validated within a single institution may not perform consistently across different populations or operational contexts, whereas broader cross-organizational collaboration can improve robustness and generalization by reducing dependence on narrowly defined local datasets [1-3].

Despite substantial investment in digital health and AI, many initiatives that show promising results in pilot settings do not progress to sustainable operational services [4,5]. Explanations commonly emphasize model performance, data quality, clinical validation, and local implementation barriers, but operationalization may also require the collaboration itself to change. Adding an organization can entail further contracting, governance review, technical integration, and assurance, and large-scale health-data programs have identified organizational and technical integration as important sources of delay [6].

Pilots can usually operate within a predefined collaborative arrangement in which participating organizations, datasets, analytical objectives, and responsibilities are established before execution and remain relatively stable. Operational services must accommodate changes in participation, available data, and analytical capabilities as needs and circumstances evolve. Scaling concerns not only the execution of distributed computation but also the ability of a governed collaboration to evolve without reconstructing its governance and technical arrangements for each new activity. Recent system-level evidence from the National Health Service AI Lab similarly identifies the pilot-to-scale gap, with fragmented governance and lack of system orchestration among the mutually reinforcing mechanisms constraining the scaling of health care AI [7]. In this study, scalability refers to the capacity of the governed collaboration to accommodate changes in participants, resources, and operations without altering its governance conditions. Computational throughput and deployment scale are outside this definition.

Existing health-data infrastructures address important parts of this problem at different boundaries. Centralized platforms provide common custody and access mechanisms, trusted research environments (TREs) provide controlled analytical access to governed datasets, federated learning (FL) frameworks coordinate computation across organizational boundaries, keeping raw data local, and dataspace or policy frameworks establish requirements for participation and data sovereignty. Their functions remain necessary, yet the decision that a participant may enter a particular federated activity under a defined authority, purpose, and set of capabilities can remain distributed across contracts, accreditation, identity systems, application configuration, and operational controls.

Open Health addresses the transition from fixed collaboration to evolving service through federated computing (FC), which represents collaboration as a governed structure within which autonomous organizations retain sovereignty over local data and systems for participating under shared governed rules [8,9]. FC complements health-data platforms, identity systems, FL frameworks, and organization-local security controls by providing the governance structure through which participation becomes legitimate within an existing federation.

Session-bound admission is one mechanism for establishing such participation [10]. Admission is evaluated for a specific governed activity, purpose, and bounded set of capabilities. Different participation relations can coexist within the same federation. An organization may, for example, submit locally computed model updates to federated training, without exposing its underlying local data, under a bounded contribution relation while retaining a status distinct from existing federation members. Contribution and model consumption are governed through separate capabilities, where a capability denotes an explicitly granted permission to perform a specified federation operation. Collaboration growth can proceed through purpose-specific and asymmetric participation relations, preserving the governance conditions of the federation.

The Open Health demonstrator examines this proposition in a multisite FL setting using Pathology Modified National Institute of Standards and Technology (PathMNIST) [11,12]. PathMNIST provides a biomedical benchmark derived from colorectal histopathology, but the federation-level partitioning used in this study is experimentally constructed and is not intended to reproduce the full clinical, organizational, or technical heterogeneity of operational health institutions.

An initial federation comprising hospitals A and B is extended through the governed participation of hospital C as a sponsored contributor. Hospital C may contribute to federated training but may not consume the resulting model only by virtue of that contribution. The implementation enables examination of whether federation participation can expand and preserve explicit authority, capability boundaries, session binding, and auditable decision evidence. This study, therefore, addresses the following research question: Can an open federated health data collaboration support governed evolution through new forms of participation and preserve bounded capabilities and auditable decision evidence?

The paper makes 3 contributions: First, it reframes the transition from pilot to service as a problem of governed federation evolution beyond computational or data scale. Second, it presents an Open Health implementation of FC in which session-bound admission, capability separation, and auditable decision evidence are integrated with a conventional FL runtime. Third, it evaluates governed federation evolution through controlled organizational and computational participation scenarios, together with the analytical consequence and admission overhead of the implemented mechanisms.

The remainder of the paper is organized as follows: the Methods section reviews existing approaches to collaborative health-data infrastructure and their governance boundaries, introduces the FC and federation-admission principles used by Open Health, and describes the system implementation, experimental scenarios, and evaluation criteria. The Results section reports the admission, analytical, AI-agent conformance, and admission-overhead results. The Discussion section discusses the principal findings, their relationship to existing health-data infrastructure, the role of decision evidence and governed AI-agent participation, and the limitations and directions for future work.

### Existing Solutions and Related Work

#### Collaborative Health-Data Infrastructures

Health-data collaboration is supported by several classes of infrastructure, including centralized data platforms, TREs, proprietary federated platforms, open-source FL frameworks, and policy or data-sovereignty frameworks. They are not direct substitutes because each establishes a different boundary around data custody, computation, participation, and governance.

Centralized data platforms simplify integration by bringing data under a common technical and administrative environment, where repository-level identity, access control, and data-management mechanisms can govern users and datasets [13,14]. Such an arrangement is effective when common custody is organizationally and legally acceptable but is less suitable when participating organizations must retain direct sovereignty over sensitive health data. TREs adopt a different boundary by providing controlled environments in which approved researchers or projects analyze specified datasets under explicit governance and disclosure controls. OpenSAFELY (Bennett Institute for Applied Data Science) [15] is a prominent example. TREs provide strong mechanisms for research approval, controlled access, and accountability, principally governing access to approved data and projects. The evolution of a federation of autonomous organizations and operational relationships constitutes a separate governance problem.

Proprietary integration and federated platforms, including Rhino Health [16], Syntropy [17], and the Mayo Clinic Platform [18], support cross-site analytics allowing participating organizations to retain local data. Their governance, deployment, and operational mechanisms are defined within the corresponding platform environment. Open-source FL frameworks such as Flower (Flower Labs GmbH) [19], NVIDIA FLARE (NVIDIA Corporation) [20], Intel OpenFL [21], PySyft (Andrew Trask) [22], and Substra (Owkin) [23] similarly provide computational mechanisms for coordinating distributed machine-learning workflows, keeping raw data local. In both cases, FC can be technically coordinated, and authority to participate remains represented through platform governance, deployment configuration, or surrounding organizational processes. Hybrid cloud architectures provide another mechanism for retaining organizational control over sensitive data for performing distributed computation. Brown and Randall [24] evaluated a secure record-linkage architecture in which identifiable information remained on premises, but privacy-preserving computation was performed in cloud infrastructure. Open Health addresses a complementary boundary by governing whether and under what conditions a participant may invoke a federated activity, independently from where the computation is executed.

Cross-organizational collaboration also depends on semantic interoperability because participating institutions may represent clinically equivalent concepts through different data structures, terminologies, coding systems, or information models. Rosenau et al [25] developed an intermediate query representation to bridge heterogeneous repository-specific query languages within a federated German health research infrastructure. Shared vocabularies and ontologies can make distributed data interpretable across organizational boundaries. Entitlement to contribute a resource, consume a resulting model, or perform an operation under a particular authority is established separately through federation governance. Semantic interoperability and federation governance address complementary requirements.

Policy-oriented dataspace initiatives, including Gaia-X [26], the European Health Data Space (EHDS) [27], and Towards the European Health Data Space [28], address trustworthy exchange, organizational eligibility, interoperability, and data sovereignty at the ecosystem level. Their emphasis on organizational control is closely aligned with cross-organizational health-data collaboration. Conversely, an operational federation-level admission decision cannot replace the regulatory, contractual, ethical, or organizational conditions that established eligibility.

AI-agent frameworks introduce a further implementation layer by coordinating how computational participants invoke tools, services, workflows, memory, or other runtime resources. Tool restrictions, isolation, approval mechanisms, and monitoring can constrain execution, but such controls do not determine whether an AI-driven process is entitled to participate in a federated health-data activity, under whose authority it acts, or which federation capabilities it may exercise. Open Health treats the agent framework as an execution mechanism whose implementation may vary independently of the federation’s governance semantics.

#### Governance Boundaries in Cross-Organizational Collaboration

Cross-organizational infrastructures depend on established identity and access-control mechanisms. Federated identity systems can authenticate principals across organizational boundaries. Open authorization (OAuth)–based mechanisms, cloud identity and access management (IAM), role-based access control (RBAC), and attribute-based access control (ABAC) can control access to services and resources [29-32]. Such technologies provide necessary deployment foundations by establishing whether an identity can be trusted, whether a service account may invoke an API, or whether an actor possessing specified attributes may access a resource. Participation in a shared activity raises a different question because the relevant decision must be established across organizational boundaries.

In operational deployments, the effective participation decision may be distributed across cloud IAM policies, platform configuration, central policy services [33-35], contractual arrangements, and checks embedded in orchestration code. Heterogeneous identity and infrastructure stacks can make integration increasingly bespoke as collaborations evolve, but configuration changes can alter effective participation semantics and complicate reconstruction of earlier decisions. Organization-local access control and cross-organizational admission address different governance boundaries. The former controls activity within an administrative domain, whereas the latter establishes whether a participant may enter a specified collaborative activity under the applicable federation conditions.

Cross-silo FL illustrates the separation between computational coordination and participation governance. A federated learning (FL) framework can coordinate model distribution, local training, update submission, aggregation, and evaluation, leaving the authority for participation external to the learning procedure. Prior work on auditable session admission has shown that requests can be evaluated against session-specific capabilities and holder identity before reaching the FL runtime, producing explicit ALLOW or DENY decisions and structured decision evidence [10]. The underlying training and aggregation procedure remains unchanged. Open Health adopts the separation between governance boundary and analytical runtime, applying it to the broader problem of federation evolution.

Table 1 summarizes the principal approaches according to the boundary at which governance is exercised, and the extent to which changes in participation become explicit operational decisions. The comparison is functional: the different approaches may coexist within the same health-data infrastructure. Open Health introduces a complementary boundary at the transition from institutional eligibility or agreement to participation in a particular governed federated activity. Authentication, regulatory or dataspace eligibility, FL-platform interoperability, and valid local permissions establish the necessary preconditions. Entitlement to a particular federation activity is determined separately through federation admission for the specified purpose and bounded capability. Open Health addresses that transition by making the participation decision explicit, session-bound, and auditable, leaving data custody, local identity, operational authorization, semantic interoperability, and analytical execution to the mechanisms suited to those functions.

**Table 1.** Functional comparison of approaches supporting cross-organizational health-data collaboration.

Approach	Primary governance boundary	Data remain under organizational control	Controls operational access	Establishes purpose-specific participation	Participation decision externally auditable	Supports governed change of participation
Centralized data platforms: enterprise cloud data lakes [14]	Repository or data controller	Usually no	Yes	Deployment dependent	Deployment dependent	Through repository governance
TREs^a^ [15,36,37]	Approved project or research environment	Often logically controlled by custodian	Yes	Yes, for approved research	Yes	Primarily through new project or governance approval
Proprietary federated platforms [16-18]	Platform federation	Yes	Yes	Platform dependent	Platform dependent	Platform dependent
Open-source FL^b^ frameworks [19-23]	Federated computation	Yes	Runtime dependent	Usually external to FL framework	Usually external	Usually external
Dataspace or sovereignty frameworks [26-28]	Ecosystem eligibility and exchange	Yes	Implementation dependent	At policy or ecosystem level	Governance dependent	Yes, principally at ecosystem level
Open Health or federated computing [9]	Governed federation activity	Yes	Complementary local or runtime mechanisms	Explicit and session-bound	Explicit decision evidence	Through bounded participation relations

a TRE: trusted research environment.

b FL: federated learning.

## Methods

### Dimensions of Collaboration Growth

Scaling a collaboration requires changes not only in the number or type of participants but also in the resources available to the federation, and the operations participants may perform. The 3 dimensions are related but governed through different relations. New participants establish participation relations, new datasets or models extend the federation’s resource base, and new analytical capabilities extend the set of permitted operations. Distinguishing them avoids collapsing heterogeneous entities and activities into a single category of objects to be admitted (Table 2).

**Table 2.** Dimensions of collaboration growth and their associated governance questions.

Dimension of evolution	What changes	Governance question
Participation	Organizations and human or computational participants, including AI agents	Under whose authority may they participate?
Resources	Datasets, models, compute, and analytical services	Under what conditions may they be contributed, exposed, or used?
Operations	Training, inference, validation, and analysis	Which activities are permitted, and for which purpose and scope?

Open Health treats scaling as governed evolution across participants, resources, and operations. Rights remain specific to the relation in which they are established, providing the conceptual basis for the asymmetric participation relations examined in the experimental implementation.

### FC and the Governed Federation Context

Open Health applies FC to the problem of evolving cross-organizational health-data collaboration and treats the federation as a governed relational structure. Participating organizations retain sovereignty over their local data, infrastructure, identities, and internal access mechanisms; the federation embodies the shared conditions agreed among the participating organizations under which cross-organizational participation becomes legitimate. The resulting boundary is complementary to organization-local IAM, RBAC, or ABAC, TREs, FL frameworks, and other operational controls because those mechanisms continue to govern activity within their respective administrative or computational domains.

The constitutive governance conditions originate in the agreements established among the participating organizations rather than in the capabilities of the technical implementation. A governed federation context is established before operational execution. The context identifies the authorities participating in the collaboration, the applicable constitutional and policy state, the purpose of the activity, and the conditions under which participation may occur. Open Health represents the bounded context through a federation envelope, allowing later capability and admission decisions to be interpreted relative to the governance state under which they were made. Authentication establishes identity, and prior organizational eligibility establishes eligibility to participate. Federation admission separately establishes entitlement to a specified activity.

A federated service is an operational function exposed within a governed federation and retained under the control of the organization that provides it. The service and its underlying resources remain within that organizational domain. The federation governs the relations under which participants may invoke it, including authority, purpose, capability, scope, and federation context. FL model inference, data transformation, and other analytical functions may be exposed as federated services and remain operational functions outside the federation’s constitutive governance.

FC as Code (FCaC) [9] provides the machine-processable representation through which these governance relations are expressed and evaluated. In Open Health, the governed federation context, participant authority, capabilities, purpose, scope, and related constraints are represented as executable artifacts that can be tested at the admission boundary. We separate governance establishment and capability expression from operational execution: FC models the relations governing participation, FCaC renders those relations machine-processable, admission evaluates them for a requested activity, and the corresponding federated service performs the admitted operation. Keeping these concerns distinct allows the participation structure to evolve independently of the analytical runtime and organization-local security mechanisms. Federation governance remains represented in its own constitutive artifacts, and operational configuration changes leave the conditions of participation unchanged.

FC provides a structure in which collaboration can evolve through changes in relations among participants, resources, and operations. A new organization does not need to acquire the rights of an existing member; a newly contributed resource need not become available to every participant, and permission to perform one operation need not imply permission to perform another. The relevant governance question is not whether an entity has been globally admitted to the federation, but whether a particular participation relation is legitimate within the governed context.

### Session-Bound Admission

#### Overview

Session-bound admission operationalizes the transition from an established governance context to a requested federated activity [10]. In the earlier FL-specific formulation, that governed context was exposed as a session identifier, retaining the semantics of the underlying federation envelope. Open Health makes the federation-context semantics explicit and applies the same boundary-oriented principle to participation relations that may change over time. The admission decision asks whether a specific participant may perform a specified operation, for a defined purpose and scope, within the current governed context. An admission decision with the value ALLOW or DENY is produced before the requested activity reaches the corresponding analytical or operational runtime.

Capabilities used at the admission boundary are contextual, not global. A capability issued for one governed context is not a reusable entitlement to arbitrary future activities, and possession of a capability is not equivalent to federation membership. Admission evaluates the holder, requested operation, purpose, resource, and scope against the capability and the governance state associated with the federation envelope. The analytical runtime remains responsible for executing an admitted activity. Legitimacy of the preceding participation relation is established separately at the federation boundary.

#### Admission of Participants

A participant may be a human actor or a conventional software process acting within the federation under an accountable organizational relationship. The issuer expresses the participant’s permitted federation activity through a bounded capability associated with the current federation envelope. Holder binding connects the capability to the participant for whom it was issued, while purpose, operation, resource, and scope delimit the activity that may subsequently be requested. The gatekeeper then evaluates the presented capability against the current governed context, with issuance and admission remaining separate decisions.

Separating issuance from admission supports differentiated participation. An established member may hold capabilities for both training and model use, whereas another organization may receive only a contribution capability for a specified federated training activity. Sponsorship provides an accountable route for establishing bounded participation while preserving the sponsor’s rights as a separate governance relation. Delegation and federation membership remain in separate governance relations.

The same separation applies across capability categories. Contribution and consumption are governed through separate capabilities. Admission authorizes only the specified operation and scope. Provenance similarly records where a participant, credential, model, or other artifact originates, whereas accountability concerns the authority under which participation occurs, and the party responsible for that relationship. Treating provenance and accountability as separate relations avoids inferring federation rights from technical origin or organizational association alone.

Admission decisions also produce explicit governance evidence. The evidence associates the participant, governed context, requested activity, capability, purpose, decision outcome, and reason for the decision. These boundary-generated artifacts provide the information needed to reconstruct successful and denied requests independently of application logs and historical system configurations. They also link governance intent to operational participation and preserve the decentralization of organization-local policy mechanisms.

#### Admission of AI Agents

AI agents do not require a separate constitutive governance category. From the perspective of FC, an AI agent is a form of computational participant acting under an accountable organizational relationship. The model provider, planning strategy, memory architecture, tool framework, or execution runtime may affect how the agent behaves, but none of those implementation properties independently determines its federation rights. Admission remains governed through the same relations of authority, purpose, capability, holder binding, and federation context that apply to other participants.

Agent participation nevertheless introduces an additional operational distinction between runtime capability and federation capability. An agent runtime may expose file access, network communication, tools, APIs, or other execution mechanisms. Federation entitlement is established independently through admitted capabilities. Admission authorizes the specified federation operation and scope, while local runtime and external-environment permissions remain governed by their respective controls. The federation governs boundary crossing, and the agent runtime governs how admitted execution is carried out.

Admission cannot certify that an AI agent will behave correctly after execution begins. In this study, agent containment denotes complementary runtime controls such as sandboxing, network isolation, tool policies, approval mechanisms, and monitoring that restrict what the agent can do after admission. Federation participation remains defined by the admitted capability relations, and the execution framework must not expose federation operations beyond those relations. Assurance therefore depends on conformance between the agent’s effective execution possibilities and the capabilities established by the governed admission decision. The bounded AI-agent scenario evaluates this requirement under the same federation-governance model used for human and conventional computational participants.

#### Threat and Trust Boundary

The threat model considered in this study concerns unauthorized enlargement of federation participation or capability relations. Attacks on the FL algorithm itself are outside its scope. The evaluated boundary includes capability or scope violations, holder-binding mismatch, federation-envelope mismatch, unintended propagation of rights through sponsorship or contribution, and attempts by a computational participant to exercise authority outside its admitted execution path. Model poisoning, membership inference [38], gradient leakage, model inversion [39], Byzantine aggregation, and related attacks on the analytical runtime remain outside the admission-governance mechanism evaluated here and require complementary protections at the FL and organization-local security layers [2].

### Open Health System Implementation

#### System Architecture

The Open Health demonstrator implements a governed FL environment in which participation is established before execution of the corresponding distributed computation. The architecture separates the governance and admission plane from the analytical runtime so that the conditions under which an organization or computational participant acts can be evaluated independently from the technology executing the work. Figure 1 summarizes the implementation as four interacting areas comprising the federation-authority and participant interfaces, the Open Health governance and orchestration services, the FL runtime, and the participating hospital sites. The proof of concept is implemented as a local containerized Docker deployment. The conditions the architecture must satisfy are stated independently as a conformance criterion for FCaC [40]. All cross-boundary operations in the demonstrator traverse this admission boundary before reaching the corresponding federated service. No path into a participating site bypasses it.

**Figure 1.**
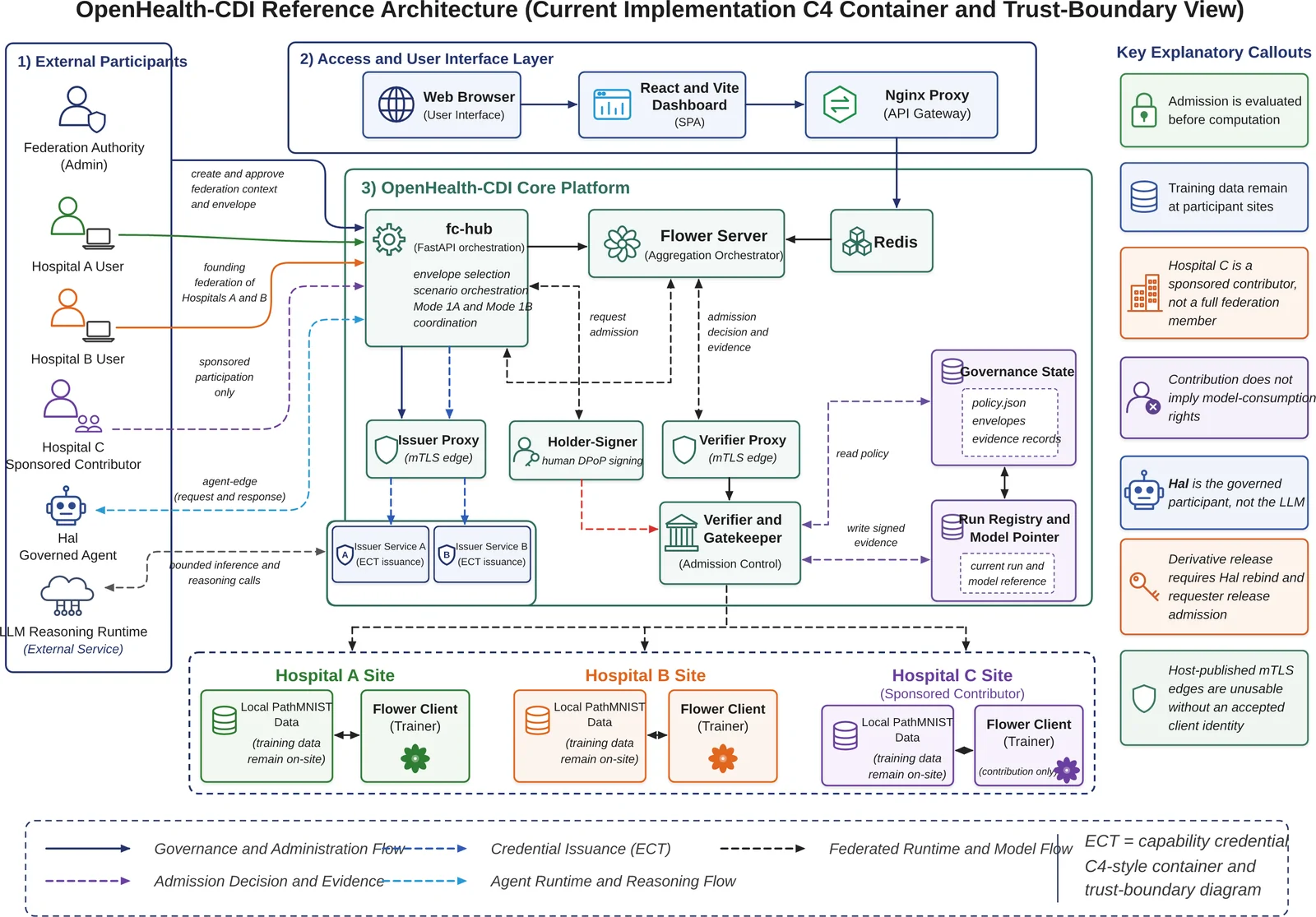
Open Health proof-of-concept architecture. The governance and admission layer establishes session-bound participation before execution by the federated learning runtime. Hospitals retain their Pathology Modified National Institute of Standards and Technology data locally. The Flower runtime exchanges model updates rather than raw data. Hospital C participates as a sponsored contributor with a capability restricted to federated training; model-consumption rights are governed separately. A bounded AI agent participates through the same governed admission path but may exercise only the capabilities granted within the active federation context. ECT: envelope capability token; LLM: large language model. C4: context, containers, components, and code; CDI: Centre for Digital Innovation;

Flower remains responsible for distributed model training and aggregation [19]. Open Health governs participation at the federation boundary. Each hospital retains its PathMNIST partition locally and exchanges only model updates through the Flower workflow, so raw images remain within the originating site. This separation confines the Open Health contribution to participation governance and leaves the learning algorithm and analytical runtime unchanged.

#### Governance and Admission Workflow

Before a federated activity begins, the federation authority establishes a governed context represented by an envelope. Creation, binding, and approval of the envelope associate the activity with the applicable constitutional and policy state. The Key Your Organization (KYO) procedure establishes the envelope through mutual transport layer security (mTLS)–authenticated organizational approvals and the required federation quorum. The resulting envelope_id is the admission binding for subsequent participation decisions and remains distinct from the operational model run created after admission [9].

Participants receive envelope capability tokens (ECTs) through the Open Health issuer service. Each ECT expresses the bounded resource, operation, purpose, and scope available to its holder and associates that capability with the governed federation context [10]. Its signature is verified against a public trust anchor associated with an issuer authorized in that context. Capability issuance, admission, and federation membership are established independently, allowing a participant to hold a narrowly scoped capability while other federation activities remain outside its authorization. For each governed request, the holder presents a request-bound demonstrating proof-of-possession (DPoP) proof signed with the key associated with the ECT. The proof binds the credential presentation to the evaluated request and establishes control of the holder key [41].

Constitutional and policy artifacts are compiled into machine-evaluable constraints for the admission boundary. The verifier and gatekeeper evaluate the ECT, holder proof, envelope_id, requested operation, purpose, resource, and scope against the current compiled governance state. Admission evaluation is stateless and fail-closed. A missing, malformed, expired, or unverifiable artifact yields DENY. No discretionary path or operator override is available at the admission boundary.

#### Runtime, State, and Decision Evidence

The web interface is built with React 18 (Meta Platforms) and Vite 7.3.5 (VoidZero), the gateway runs Nginx (F5, Inc), and the backend services are implemented with FastAPI (Sebastián Ramírez). Participant and federation-authority operations are exposed through this interface and routed through the Nginx gateway to the OpenHealth services. The fc-hub FastAPI service coordinates. The fc-hub FastAPI service coordinates the governed workflow and maintains the association between the current federation context and the operational FL run, but policy evaluation remains the responsibility of the verifier and gatekeeper. The interface and orchestration services expose governance state and invoke governed operations, while governance semantics remain defined by the federation’s constitutive artifacts.

Constitutional and policy artifacts, active federation envelopes, and admission evidence are maintained separately from the Flower runtime. Each evaluated request produces a structured decision record containing the relevant participant, governed context, requested capability, purpose and scope, decision outcome, and reason.

The run registry maintains a traceable association between the governed federation context and the model run executed by Flower, preserving them as distinct objects. Capability credentials remain bound to the federation envelope and do not extend automatically to future model runs. A decision concerning a particular executed run may record the corresponding run association when applicable. Governance context, capability, admission decision, and model run remain distinct artifacts connected through explicit relations.

#### Experimental Scenarios

The experimental design uses PathMNIST [11,12] to examine governed evolution of an initially fixed federation. Hospitals A and B establish the baseline federation. The scenarios then change the governed participation relations without changing the analytical runtime, first by admitting hospital C as a sponsored contributor and then through bounded AI-agent participation. Governance and analytical outcomes are evaluated independently. Admission correctness is established through preservation of the specified capability relations, whereas model performance is evaluated through the resulting analytical outcomes.

#### A+B Baseline

Hospitals A and B constitute the initial federation and participate as established members under the same governed federation context. Each hospital retains its local PathMNIST partition and operates a Flower client that performs local training and submits model updates to the federated aggregation process. Raw images remain within the corresponding hospital environment, and the shared model is produced through conventional federated training after the required participation has been admitted.

PathMNIST contains nine classes comprising adipose tissue, background, debris, lymphocytes, mucus, smooth muscle, normal colon mucosa, cancer-associated stroma, and colorectal adenocarcinoma epithelium. Background was excluded from the publication experiment because it represents image background rather than a histological tissue category and is therefore outside the scope of the tissue-specific analytical comparison. The active noncancer classes were adipose tissue, debris, lymphocytes, mucus, smooth muscle, and normal colon mucosa, while cancer-associated stroma and colorectal adenocarcinoma epithelium formed the cancer-related group.

The federation partition was experimentally constructed. Hospitals A and B each received 30% of the observations allocated to every active noncancer class and 5% of those allocated to each cancer-related class. Consequently, the A+B control contained 60% of the allocated observations for every active noncancer class and 10% for each cancer-related class. The remaining observations were assigned to hospital C.

Two roles of the A+B configuration are distinguished in the evaluation. The initial A+B run establishes the governance and system baseline in which the federation consists only of its existing members. For the analytical comparison, an A+B control is trained under conditions matched to the A+B+C configuration, preserving the same model architecture and learning procedure so that the comparison isolates the effect of the additional admitted contribution rather than differences in training configuration.

#### Mode 1A: Governed Expansion With a Sponsored Contributor

Hospital C is admitted to federated training as a sponsored contributor under a participation relation distinct from federation membership. Sponsorship establishes an accountable, bounded participation relation for hospital C. Its capability is restricted to the submit_update operation for the specified federated-training purpose and remains bound to the current governed federation context. Admission authorizes that specific activity while preserving hospital C’s sponsored-contributor status and the separation from member capabilities.

Model consumption is represented independently from contribution and is absent from the capability granted to hospital C. The implementation permits hospital C to contribute model updates to an admitted federated-training activity, remaining unable to query or otherwise consume the resulting model. Contribution and consumption are governed as distinct relations not inferred from participation or organizational association.

Hospital C contributed 40% of the observations allocated to each active noncancer class and 90% of the observations allocated to each cancer-related class. Accordingly, the A+B+C configuration contained the full set of allocations for every active class. Mode 1A produces two independent evaluation dimensions. The governance dimension examines whether contributions are admitted, while operations outside hospital C’s granted capability, including model consumption, remain denied. The analytical dimension compares the matched A+B control with the A+B+C model to determine whether the admitted contribution produces an observable analytical consequence.

#### Mode 1B: Bounded AI-Agent Participation

Mode 1B introduces an AI agent as a computational participant acting within the same governed federation context as the human participants. The agent has a dedicated identity and holder-bound capabilities that permit narrowly scoped inference operations but do not confer federation membership or unrestricted access to model outputs. Its execution environment is isolated from privileged governance services so that technical reachability cannot enlarge the participation relation established through admission.

The scenario considers a requester seeking cancer-related information whose unrestricted form lies outside the requester’s authorized consumption scope. Direct source consumption is therefore denied. The agent may invoke an explicitly governed rebind operation, transforming the restricted result into a policy-authorized derivative representation, which the demonstrator implements as a blurred image accompanied by a qualitative indication of model accuracy. The federation policy determines the transformation. The term rebind is retained throughout this paper in its broader operational sense of placing a derived representation under a new governance relation. This terminology emphasizes the governed outcome of the operation. In the stricter formalization of [8], the value-changing transformation V→W constitutes an Unbind, whereas Rebind preserves the value while tightening its governance envelope.

Source and derivative consumption are governed independently. The agent performs the transformation under its own admitted authority, and the requester’s access to the resulting derivative is evaluated under a separate derivative-consumption capability. The transformation and subsequent consumption remain distinct governed operations within the same federation context, preserving the distinction between the capabilities of the computational participant and those of the requesting participant.

#### AI-Agent Participation and Governance Invariants

The mode 1B design makes explicit a set of governance invariants that remain independent from the agent framework used to implement the computational participant. An agent runtime may determine how tasks are planned and tools are invoked, but the execution mechanism cannot redefine the authority under which the participant entered the federation (Table 3).

**Table 3.** Federated computing governance invariants for AI-agent participation. These invariants are the agent-participation case of the general conformance criterion for federated computing as code [40].

Governance invariant	Operational consequence for AI-agent participation
Admission is governed	Technical reachability is insufficient; the computational participant must be explicitly admitted to the requested federation activity.
Authority is explicit and bounded	The agent acts only within the authority, purpose, operation, resource, and scope represented by the admitted capability.
Sponsorship is distinct from delegation	A sponsoring organization establishes an accountable participation route; its federation capabilities remain with the sponsor.
Provenance is distinct from accountability	The agent participates under an explicitly established authority relation, independent of the origin of its model, framework, runtime, or provider.
Credentials remain bound to the federation envelope	A capability issued for one governed context remains valid only within that federation envelope and its associated activity.
Contribution and consumption are independently governed	Contribution capabilities and model-query or model-use capabilities are established as separate relations.
Execution cannot redefine governance	Tools, network paths, or runtime functions that are technically available cannot enlarge the participation relation established by the admission decision.
Derivative consumption is distinct from source consumption	A policy-authorized rebind may create a derivative that can be consumed under a capability relation different from that governing the source representation.

For an admitted computational participant, conformance requires the effective operational possibilities exposed by the execution environment to remain within the capabilities established by the governed admission decision. The criterion evaluates whether runtime behavior remains within the admitted capability boundary, including when execution is variable or fallible. It does not require the computational participant to be predictable; it makes preservation of the asserted governance boundary observable and testable even when the internal behavior of that participant varies.

### Evaluation Criteria

The demonstrator is evaluated along complementary governance and analytical dimensions. Governance behavior is assessed through positive and negative admission tests covering valid session admission, capability mismatch, holder-binding mismatch, sponsorship-based contribution, denial of model consumption to a contribution-only participant, and preservation of federation-context binding. Decision evidence is examined for the ability to associate ALLOW and DENY outcomes with the participant, requested operation and purpose, governed context, reason, and relevant model run when applicable.

- Mode 1A evaluates the analytical effect of governed federation expansion by comparing the matched A+B control with A+B+C. Cancer-related macro recall is the unweighted mean recall of classes 7 and 8. Noncancer macro recall is the unweighted mean recall of active classes 0, 2, 3, 4, 5, and 6. Class-specific recall for cancer-associated stroma and colorectal adenocarcinoma epithelium is also reported. Global macro recall and overall accuracy are reported descriptively. Analytical outcome and governance correctness are evaluated independently.
- Mode 1B evaluates conformance between the capability relations specified for the computational participant and the effective execution possibilities exposed to it. General claims about agent safety, autonomy, or model reliability fall outside the evaluation.

### Admission Overhead

Admission overhead is evaluated through a request-level microbenchmark of the admission gateway. Three decision paths are exercised: a valid ECT and holder proof with a permitted capability (ALLOW), a valid ECT and holder proof with a capability or scope mismatch (DENY-Cap), and a valid ECT presented with a mismatched holder proof (DENY-Holder). Each iteration uses a fixed valid federation context and capability credential for generating a fresh request-bound DPoP proof [41]. Per-request timings are recorded inside the admission gateway using a monotonic high-resolution clock. Measurements are retained in memory during the benchmark and written to a JavaScript Object Notation Lines (JSONL) log after the run, excluding filesystem I/O from the measured request path. The benchmark uses 1000 requests per decision path under no concurrent load, and latency is summarized as the median with the 25th and 75th percentiles.

### Ethical Considerations

This study involved no human participants or identifiable personal data. All experiments used PathMNIST, a publicly available, anonymized dataset [11,12], and hospitals A, B, and C were simulated participants. Under University College London guidance, research using anonymized public-domain datasets is minimal risk and does not require ethical approval [42], and Health Research Authority guidance generally excludes research using previously collected, nonidentifiable information from research ethics committee review [43]. Ethics review and informed consent were therefore not sought.

## Results

### Admission Behavior and Decision Evidence

The Open Health implementation produced explicit admission decisions for permitted and rejected requests at the federation boundary. In this study, evidence denotes the signed, machine-generated decision artifact emitted by the admission mechanism for each evaluated request. It is distinct from the capability credential presented by the requester, from application logs, and from the analytical artifacts produced by the FL runtime. The approach follows earlier session-admission work in treating the decision outcome, its reason, and the bindings required to reconstruct the evaluated request as first-class experimental outputs [10].

Each Open Health decision record identifies the admission event and timestamp, records the ALLOW or DENY outcome and reason, and associates the requester and holder-binding result with the relevant organizational and sponsorship information. The record also captures the requested action, purpose, and tissue scope, the governing federation envelope, the policy hash, and the related model run when applicable. Request and credential identifiers bind the decision to the specific presentation that was evaluated. An independent evidence signature protects the integrity of the resulting artifact.

The resulting evidence is independently verifiable and not merely observable. A verifier possessing the federation’s public trust anchor can check the signature and inspect the recorded outcome, reason, request bindings, and governance state independently of mutable dashboard state and historical Flower-runtime configuration. Evidence establishes the governance decision and the conditions under which it was made. Clinical correctness of the subsequent analytical result is evaluated separately (Table 4).

**Table 4.** Principal elements of an Open Health admission-evidence record.

Evidence element	Representative content	Audit role
Decision	Decision identifier, timestamp, ALLOW or DENY, and reason	Identifies the outcome produced at the admission boundary and why it was reached.
Principal and accountability	Subject, actor type, issuing organization, sponsorship relation, and requester-binding result	Associates the decision with the participant and the authority relation under which the request was made.
Requested activity	Operation, purpose, resource, and requested tissue scope	Records the activity against which the presented capability was evaluated.
Governed context	Federation envelope, policy hash, and related model run when applicable	Binds the decision to the governance state and operational context in which it was valid.
Request and credential binding	Request identifier and capability-credential fingerprint	Distinguishes the evaluated presentation from other requests or credentials.
Integrity	Signed decision artifact and evidence-key identifier	Allows later verification that the recorded decision has not been modified.

Positive and negative tests were used to determine whether the admission boundary preserved the capability relations specified by the federation. Table 5 summarizes the principal cases. Ordinary member participation, hospital C’s sponsored contribution, attempted model consumption outside hospital C’s capability, scope violations, holder-binding failure, and federation-envelope mismatch are evaluated independently. Each case produces its own decision record. Each DENY is recorded as an explicit governance event.

**Table 5.** Admission and capability-conformance results.

Test condition	Expected	Observed	Decision evidence
Established member requests an operation within the admitted capability	ALLOW	ALLOW	Capability match
Hospital C submits an update for the admitted federated training purpose	ALLOW	ALLOW	Capability match under sponsored contribution
Hospital C requests model consumption outside its granted capability	DENY	DENY	Capability violation
Requested pathology scope exceeds the capability scope	DENY	DENY	Capability scope exceeded
Presented proof does not match the credential holder binding	DENY	DENY	Holder-binding mismatch
Presented capability is bound to a different federation envelope	DENY	DENY	Envelope mismatch

### Governed Expansion From A+B to A+B+C

The A+B configuration established the initial federation with 2 existing members participating in federated training under the same governed context. Mode 1A then introduced hospital C through sponsorship, preserving the status and capabilities of hospitals A and B and leaving the Flower training and aggregation procedures unchanged. Hospital C was admitted for the submit_update operation associated with the federated-training purpose, and the corresponding admission evidence remained associated with the governed context and executed model run.

The complementary negative test preserved the asymmetric relation established for hospital C. A request to query or otherwise consume the resulting model was denied because model consumption was absent from the sponsored contributor’s capability set. Successful contribution combined with unsuccessful model consumption provides the primary governance result for mode 1A. Federation growth occurred by adding a bounded participation relation instead of promoting hospital C to an equivalent member or transferring the capabilities of its sponsor.

### Analytical Effect of the Additional Contribution

PathMNIST model performance was measured to verify that hospital C’s admitted contribution produced an observable analytical consequence and not a purely administrative change. The analytical comparison uses an A+B control trained under conditions matched to the A+B+C run. An earlier A+B baseline used during development is not treated as the comparative reference because its training duration differs from that of the governed-expansion experiment (Table 6).

**Table 6.** Matched analytical comparison between A+B control and governed A+B+C expansion.

Metric	A+B control (%)	A+B+C expansion (%)	Change (pp)^a^
Cancer-related macro recall	50.2	67.4	+17.2
Noncancer macro recall	85.5	86.3	+0.8
Cancer-associated stroma recall	19.5	39.9	+20.4
Colorectal adenocarcinoma epithelium recall	80.9	94.9	+13.9
Global macro recall	76.7	81.6	+4.9
Overall accuracy	82.0	86.4	+4.5

a pp: percentage points.

Admission of hospital C increased cancer-related macro recall from 50.2% to 67.4% (+17.2 percentage points), while noncancer macro recall remained similar at 85.5% and 86.3%, respectively. Within the cancer-related classes, recall increased from 19.5% to 39.9% for cancer-associated stroma and from 80.9% to 94.9% for colorectal adenocarcinoma epithelium. Global macro recall increased from 76.7% to 81.6%, and overall accuracy from 82.0% to 86.4%.

These analytical results establish that hospital C’s admitted contribution had an observable effect on the workload. Governance correctness is established separately through preservation of the admitted capability relations and their signed admission records. Model-performance improvement is therefore not treated as evidence that admission was correctly governed.

### AI-Agent Conformance and Rebind

Mode 1B introduces an AI agent as a computational participant with its own identity and holder-bound capabilities. A requesting participant may lack authority to consume an unrestricted source result. Direct consumption of the restricted source must remain denied to a requester lacking the corresponding source-consumption capability.

The requester is not authorized to consume the unrestricted cancer-related source result. The agent may nevertheless perform bounded inference and invoke the governed rebind operation, which converts the restricted result into a policy-authorized derivative consisting of a blurred tissue image and a qualitative indication of prediction accuracy. The requester may consume this derivative only under a separate derivative-consumption capability. The agent performs the transformation under its own admitted authority, and federation policy determines the permitted transformation. The Mode 1B scenario follows the conformance requirements summarized in Table 7, which define the required decisions across source consumption, bounded inference, policy-authorized rebind, derivative consumption, and access to privileged governance paths.

**Table 7.** Mode 1B conformance requirements and observed decisions for bounded AI-agent participation.

Governed operation	Required decision	Observed decision	Governance requirement
Requester asks for unrestricted cancer-related source output	DENY	DENY	Source-consumption capability remains absent
The agent performs inference within its bounded capability	ALLOW	ALLOW	Agent authority remains explicit and holder-bound
The agent invokes the policy-authorized rebind operation	ALLOW	ALLOW	Transformation is determined by federation policy
Requester consumes the derived representation under the corresponding capability relation	ALLOW	ALLOW	Derivative consumption is evaluated independently from source consumption
The agent attempts a privileged governance path outside the admitted execution route	DENY	DENY	Execution cannot enlarge federation authority

### Admission Overhead

Admission adds cryptographic verification, capability matching, evidence signing, and evidence persistence to the request path. Table 8 reports the completed request-level benchmark for the current Open Health implementation.

**Table 8.** Server-side admission latency by decision path.

Operation	ALLOW^a^, median (IQR) ms^b^	DENY-Cap^c^, median (IQR) ms	DENY-Holder^d^, median (IQR) ms
ECT^e^ verification	0.150 (0.143‐0.171)	0.148 (0.143‐0.160)	0.150 (0.144‐0.161)
DPoP^f^ verification	0.112 (0.110‐0.120)	0.114 (0.111‐0.120)	0.144 (0.139‐0.151)
Capability match	0.0051 (0.0048‐0.0053)	0.0051 (0.0049‐0.0054)	NA^g^
Full admission check	1.210 (1.141-1.333)	1.195 (1.136‐1.273)	0.884 (0.837‐0.921)

a ALLOW denotes a valid capability and holder proof.

b Values are median (IQR) ms, from 1000 requests per case.

c DENY-Cap denotes a capability or scope mismatch.

d DENY-Holder denotes a holder-binding mismatch.

e ECT: envelope capability token.

f DPoP: demonstrating proof of possession.

g NA indicates that capability matching was not reached because holder validation failed first.

Median full admission latency was 1.210 (IQR 1.141‐1.333) ms for successful admission, 1.195 (IQR 1.136‐1.273) ms for capability-scope denial, and 0.884 (IQR 0.837‐0.921) ms for holder-binding denial. ECT verification, DPoP verification, and capability matching individually remained submillisecond. Capability matching is reported as not applicable for the holder-binding denial because the request fails closed during DPoP verification and evaluation terminates before the capability-matching stage. The shorter full latency for this path is consistent with that early termination. For ALLOW, decision-evidence signing and persistence had median latencies of 0.065 (IQR 0.063-0.069) ms and 0.164 (IQR 0.159-0.172) ms, respectively, with comparable values for the denial paths.

The full admission measurement includes cryptographic verification, capability evaluation, decision-evidence signing and persistence, and the surrounding admission-gateway processing. The benchmark is intended as a deployability check rather than a throughput study and reports single-request latency under no concurrent load. The relevant systems question is whether explicit admission and signed decision evidence introduce material overhead relative to the federated activity they govern; throughput and tail latency under concurrent requests remain outside the current evaluation.

## Discussion

### Principal Findings

The Open Health demonstrator supports the central proposition of the study by representing federation growth as a governed change in participation relations, with technical onboarding remaining subject to explicit governance conditions. Positive and negative admission decisions remain tied to the federation envelope and are retained as signed evidence. Mode 1B extends the same structure to computational participation by separating the agent’s execution capability from the rights of the human participant requesting the result.

Model accuracy is a secondary outcome in the current evaluation. Under matched training conditions, cancer-related macro recall increased from 50.2% to 67.4% after hospital C’s admitted contribution, while noncancer macro recall remained similar at 85.5% and 86.3%. This establishes an observable analytical consequence of the governed contribution but neither establishes nor measures governance correctness. The primary result is preservation of the capability relations asserted before execution, including contribution-specific capabilities, holder-bound admission, federation-context binding, and independent governance of source and derivative consumption.

### From Fixed Collaboration to Governed Federation Evolution

Many collaborative infrastructures are designed around an initially established set of organizations, resources, and analytical operations. Their technical mechanisms may scale effectively once those assumptions are fixed, yet movement from pilot to operational service can require the collaboration itself to change. Open Health addresses that transition by treating participation, resources, and operations as separable dimensions whose relations can evolve under an existing governance context.

The concept of a federated service follows from the same separation. An operational function can be exposed within the federation while remaining under the control of its provider, with the federation governing the authority, purpose, capability, scope, and context under which it may be invoked. Flower-based training and inference provide the concrete services used in the demonstrator. The governance structure is runtime-independent and can in principle accommodate other analytical or data-processing services as operational functions outside the federation’s constitutive layer.

### Relationship to Existing Health-Data Infrastructure

Open Health is not intended to replace TREs, identity federation, IAM, RBAC, or ABAC, dataspace governance, or FL frameworks. Each addresses a different boundary. Organization-local controls remain responsible for identities, infrastructure, users, and resources within an administrative domain, while analytical runtimes remain responsible for executing admitted computation. The additional FC boundary establishes whether a participant may enter a particular federated activity under the current shared governance conditions.

The distinction becomes important when collaborations evolve asymmetrically. Conventional onboarding may be sufficient when every new organization is intended to receive the same role and operational privileges as existing members. Hospital C represents a different case because the desired relation is deliberately narrower. Sponsorship enables accountable, differentiated participation while preserving delegation and membership as separate governance relations.

### AI-Agent Participation and Governed Transformation

Mode 1B illustrates that an AI agent fits within the existing constitutive categories of the federation. The agent acts as a computational principal with its own identity, holder binding, and bounded capabilities. Its planning strategy or runtime determines how an admitted operation is executed. Runtime isolation constrains execution paths; federation authority is established through governed admission. Rebind extends this structure with an additional governed relation.

The agent may transform a restricted result into a policy-authorized derivative; the recipient’s rights to that derivative are evaluated independently from rights to the source. The blurred representation used in the demonstrator is not treated as a differential-privacy mechanism or as an agent-defined judgment that an output is safe. Its significance lies in the governed transformation and the independent capability relation attached to subsequent consumption. The construction provides an operational connection to the earlier value-identity model, in which transformations of data and their governance bindings are considered jointly [8].

The same layering limits the claim that can be made about agent safety. Admission establishes legitimate participation at a federation boundary, whereas sandboxing, tool restrictions, network isolation, monitoring, and approval mechanisms constrain behavior within the execution environment. Neither layer can substitute for the other. The Open Health result concerns conformance between federation admission and runtime execution; agent predictability lies outside the scope of the study.

### Limitations and Future Work

The demonstrator is a proof of concept implemented outside a production health-data infrastructure. PathMNIST provides a biomedical benchmark for reproducibly exercising governance relations and class-specific analytical effects. Clinical effectiveness and performance across heterogeneous operational health systems remain subjects for subsequent evaluation. The current proof of concept includes 3 participating hospitals and focuses on technical federation governance. Institutional processes required to establish real cross-organizational agreements remain outside its scope. The implementation will be ported to Amazon Web Services as part of the University College London Centre for Digital Innovation Impact Accelerator Program beginning in September 2026. The resulting cloud deployment will provide an environment for evaluating operational deployment and scaling and for verifying preservation of the governance invariants examined in this study.

The admission benchmark characterizes single-request latency in the current implementation and deployment environment under no concurrent load and does not establish throughput, tail-latency, or scale-out performance. Key rotation, revocation, recovery, long-duration policy evolution, and operational incident handling require further evaluation. History-dependent controls such as cumulative disclosure limits belong to a procedural layer beyond the per-request admission mechanism evaluated here.

Mode 1B is restricted to a deliberately bounded agent interaction, with arbitrary autonomous behavior, unrestricted tool use, and unsupervised clinical decision-making outside its scope. Further work should examine multiagent and multisponsor participation, where computational participants operate under distinct accountable sponsorship relations and interact through bounded federation capabilities, with authority remaining specific to each participant. Deployments across different analytical frameworks and health-data environments would also test whether the governance boundary remains portable when the underlying execution substrate changes.

### Conclusion

Open Health reframes the transition from federated pilot to operational service as a problem of governed federation evolution. The demonstrator separates the establishment of a federation context, expression of bounded capabilities, admission of specific participation, execution by a conventional analytical runtime, and production of signed decision evidence. Federation growth can occur through differentiated participation relations that preserve membership boundaries and bounded capability relations.

The hospital C scenario demonstrates contribution under a dedicated capability, with model consumption governed separately. The AI-agent scenario extends the same semantics to a computational participant producing a policy-authorized derivative under its own admitted capability, while recipient access remains independently governed. Together, the scenarios show how participants and operations can evolve while preserving explicit authority and capability boundaries.

The principal contribution is not a new FL algorithm or a claim of improved clinical model performance. It is a machine-evaluable admission boundary through which an existing collaboration can change while retaining auditable evidence of the conditions under which participation was allowed or denied. Such a boundary provides one route from fixed federated health-data demonstrations to services that can evolve while preserving organizational sovereignty.

## Abbreviations

ABAC: attribute-based access control
DPoP: demonstrating proof-of possession
ECT: envelope capability token
EHDS: European Health Data Space
FC: federated computing
FCaC: federated computing as code
FL: federated learning
RBAC: role-based access control
TRE: trusted research environment
